# The complete chloroplast genome sequence of *Phoebe puwenensis*

**DOI:** 10.1080/23802359.2019.1699469

**Published:** 2019-12-12

**Authors:** Jinfeng Zhang, Yunqing Li, Yi Wang

**Affiliations:** Laboratory of Forest Plant Cultivation and Utilization, Yunnan Academy of Forestry, Kunming Yunnan, People’s Republic of China

**Keywords:** *Phoebe puwenensis*, chloroplast, Illumina sequencing, phylogenetic analysis

## Abstract

The first complete chloroplast genome (cpDNA) sequence of *Phoebe puwenensis* was determined from Illumina HiSeq pair-end sequencing data in this study. The cpDNA is 152,746 bp in length, contains a large single-copy region (LSC) of 93,685 bp and a small single-copy region (SSC) of 18,909 bp, which were separated by a pair of inverted repeats (IR) regions of 20,076 bp. The genome contains 127 genes, including 82 protein-coding genes, 8 ribosomal RNA genes, and 36 transfer RNA genes. The overall GC content of the whole genome is 39.1%, and the corresponding values of the LSC, SSC, and IR regions are 37.9%, 33.9%, and 44.4%, respectively. Further phylogenomic analysis showed that *P. puwenensis* and *Phoebe neurantha* clustered in a clade in Phoebe genus.

*Phoebe puwenensis* is the species of the genus *Phoebe* within the family Lauraceae (Zhu et al. 2005). It is distributed in the evergreen broad-leaved forest of Southern Yunnan (Liu et al. 2018). *Phoebe puwenensis* is a precious timber tree in the hot area (Zhu 2004). Its wood can be used for construction, furniture, farm tools, and it has huge economic value (Cui et al. 2015). However, there has been no genomic studies on *P. puwenensis*.

Herein, we report and characterize the complete *P. puwenensis* plastid genome (MN698968). One *P. puwenensis* individual (specimen number: 5309270591) was collected from Cangyuan, Yunnan Province of China (23°17′40ʺN, 99°11′16ʺE). The specimen is stored at Yunnan Academy of Forestry Herbarium, Kunming, China, and the accession number is YAFH0012764. DNA was extracted from its fresh leaves using DNA Plantzol Reagent (Invitrogen, Carlsbad, CA, USA).

Paired-end reads were sequenced using Illumina HiSeq system (Illumina, San Diego, CA). In total, about 21.1 million high-quality clean reads were generated with adaptors trimmed. Aligning, assembly, and annotation were conducted by CLC *de novo* assembler (CLC Bio, Aarhus, Denmark), BLAST, GeSeq (Tillich et al. 2017), and GENEIOUS v 11.0.5 (Biomatters Ltd, Auckland, New Zealand). To confirm the phylogenetic position of *P. puwenensis*, other five species of *Phoebe* genus from NCBI were aligned using MAFFT v.7 (Katoh and Standley 2013). The Auto algorithm in the MAFFT alignment software was used to align the six complete genome sequences and the G-INS-i algorithm was used to align the partial complex sequences. The maximum likelihood (ML) bootstrap analysis was conducted using RAxML (Stamatakis 2006); bootstrap probability values were calculated from 1000 replicates. *Cinnamomum camphora* (MH356726) and *Cinnamomum bodinieri* (MH394416) were served as the out-group.

The complete *P. puwenensis* plastid genome is a circular DNA molecule with the length of 152,746 bp, contains a large single-copy region (LSC) of 93,685 bp and a small single-copy region (SSC) of 18,909 bp, which were separated by a pair of inverted repeats (IR) regions of 20,076 bp. The overall GC content of the whole genome is 39.1%, and the corresponding values of the LSC, SSC, and IR regions are 37.9%, 33.9%, and 44.4%, respectively. The plastid genome contained 127 genes, including 82 protein-coding genes, 8 ribosomal RNA genes, and 36 transfer RNA genes. Phylogenetic analysis showed that *P. puwenensis* and *Phoebe neurantha* clustered in a unique clade in *Phoebe* genus (Figure 1). The determination of the complete plastid genome sequences provided new molecular data to illuminate the *Phoebe* genus evolution.

**Figure 1.**
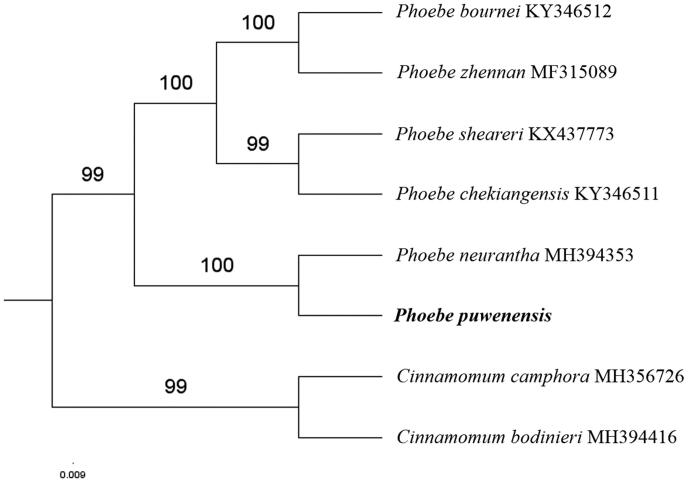
The maximum-likelihood tree based on the six chloroplast genomes of *Phoebe* genus. The bootstrap value based on 1000 replicates is shown on each node.
